# Considerations for the treatment of pancreatic cancer during the COVID-19 pandemic: the UK consensus position

**DOI:** 10.1038/s41416-020-0980-x

**Published:** 2020-07-08

**Authors:** Christopher M. Jones, Ganesh Radhakrishna, Katharine Aitken, John Bridgewater, Pippa Corrie, Martin Eatock, Rebecca Goody, Paula Ghaneh, James Good, Derek Grose, Daniel Holyoake, Arabella Hunt, Nigel B. Jamieson, Daniel H. Palmer, Zahir Soonawalla, Juan W. Valle, Maria A. Hawkins, Somnath Mukherjee

**Affiliations:** 1 Faculty of Biological Sciences, University of Leeds, Leeds, UK; 2 Radiotherapy Research Group, Faculty of Medicine and Health, University of Leeds, Leeds, UK; 3 Leeds Cancer Centre, The Leeds Teaching Hospitals NHS Trust, Leeds, UK; 4 The Christie NHS Foundation Trust, Manchester, UK; 5 The Royal Marsden Hospital, The Royal Marsden NHS Foundation Trust, London, UK; 6 The Institute of Cancer Research, London, UK; 7 University College London Cancer Institute, London, UK; 8 Department of Oncology, Cambridge University Hospitals NHS Foundation Trust, Cambridge, UK; 9 The Northern Ireland Cancer Centre, Belfast, UK; 10 The Royal Liverpool University Hospital, Liverpool, UK; 11 University Hospitals Birmingham NHS Foundation Trust, Birmingham, UK; 12 Beatson West of Scotland Cancer Centre, Glasgow, UK; 13 Norfolk and Norwich University Hospitals NHS Foundation Trust, Norwich, UK; 14 Wolfson Wohl Cancer Research Centre, University of Glasgow, Glasgow, UK; 15 The Clatterbridge Cancer Centre NHS Foundation Trust, Liverpool, UK; 16 Liverpool Experimental Cancer Medicine Centre, University of Liverpool, Liverpool, UK; 17 Oxford University Hospitals NHS Foundation Trust, Oxford, UK; 18 Division of Cancer Sciences, University of Manchester, Manchester, UK; 19 Department of Medical Physics and Biomedical Engineering, University College London, London, UK; 20 CRUK/MRC Oxford Institute for Radiation Oncology, University of Oxford, Oxford, UK

**Keywords:** Pancreatic cancer, Radiotherapy, Chemotherapy

## Abstract

The coronavirus disease 2019 (COVID-19) pandemic epicentre has moved to the USA and Europe, where it is placing unprecedented demands on healthcare resources and staff availability. These service constraints, coupled with concerns relating to an increased incidence and severity of COVID-19 among patients with cancer, should lead to re-consideration of the risk–benefit balance for standard treatment pathways. This is of particular importance to pancreatic cancer, given that standard diagnostic modalities such as endoscopy may be restricted, and that disease biology precludes significant delays in treatment. In light of this, we sought consensus from UK clinicians with an interest in pancreatic cancer for management approaches that would minimise patient risk and accommodate for healthcare service restrictions. The outcomes are described here and include recommendations for treatment prioritisation, strategies to bridge to later surgical resection in resectable disease and factors that modify the risk–benefit balance for treatment in the resectable through to the metastatic settings. Priority is given to strategies that limit hospital visits, including through the use of hypofractionated precision radiotherapy and chemoradiotherapy treatment approaches.

## Background

Following the first reports of infection with severe acute respiratory syndrome coronavirus 2 during December 2019 in Wuhan, China, cases of coronavirus disease 2019 (COVID-19) have dramatically increased across the world.^1^ With its epicentre now in Europe and the USA, the COVID-19 pandemic is placing unprecedented demands on healthcare resources across a number of countries. This includes the United Kingdom (UK), where increasing numbers of patients critically unwell from COVID-19 have in some areas severely diminished bed availability within high-dependency and intensive care units, reducing surgical capacity as a consequence. A reduction in the numbers of frontline healthcare workers through infection and self-isolation is also increasing service pressures.

Adding further challenge to standard cancer treatment pathways, a majority of patients with cancer are immunosuppressed and may be more likely to contract COVID-19.^2–6^ Given that hospitals act as a reservoir for infection, this risk is amplified by multiple hospital attendances for cancer diagnosis, treatment and follow-up. A cancer diagnosis and recent anticancer treatment may additionally be linked to greater severity of COVID-19.^2–6^ As such, the risk–benefit balance is likely to have changed for a number of cancer treatments, although it should be noted that evidence of the magnitude of risk conferred by COVID-19 for patients with cancer, and for those receiving anticancer therapies, remains uncertain.^7^ Adding further complexity, cancer services must now forward plan for possible recurrent peaks in COVID-19 incidence while managing the lasting consequences of the first outbreak. This includes both a backlog of cases resulting from the clear pivot of the National Health Service (NHS) towards a focus on COVID-19 treatment and, in some areas, continuing to grapple with a prolonged plateau in first peak COVID-19 incidence.

In light of this, we convened an expert group of UK clinicians with expertise in pancreatic cancer. The panel identified areas in which resource limitations or the potential for SARS-CoV-2 infection would potentially increase the risks of, or limit access to, current standard treatments for pancreatic cancer. This included a review of guidance relating to COVID-19 published by NHS England and other relevant UK professional bodies. Alternative management strategies for these scenarios were sought via literature review and through input from panel members. Identified options were virtually reviewed by the panel and used to formulate an initial guidance document. This subsequently received iterative input from the panel until consensus was reached, with a focus throughout on management approaches that would minimise risk to the patient and accommodate for healthcare service restrictions, such as through where possible limiting hospital attendance in line with the RADS (Remote, Avoid, Defer, Shorten) principle.^8,9^ The 18-member panel, which included surgeons, clinical (radiation) oncologists and medical oncologists, are listed in Supplementary information. Additional feedback was received from patient and public representatives via Pancreatic Cancer UK, a registered pancreatic cancer charity.

The proposals developed through this process are summarised in Table 1 and have been revised as the COVID-19 outbreak has evolved. They should serve to guide clinicians both as the initial COVID-19 peak plateaus and resolves, and in any subsequent disease outbreaks. These should be considered in conjunction with other documents outlining stratification and prioritisation of surgery, chemotherapy and radiotherapy (RT) delivery during the COVID-19 pandemic.^10–15^

**Table 1:** Suggested approaches for and key points relating to the management of patients with pancreatic cancer during the COVID-19 pandemic.

General principles
• The risks conferred by COVID-19 are greater for older patients and those with comorbidities. • Minimise hospital visits, including through use of telephone consultations. • Educate patients regarding the importance of physical distancing measures. • Use hypofractionated regimes where radiotherapy is to be delivered. • Dose modification and the use of prophylactic growth factor and antibiotics may mitigate SACT risks. • Treatment decisions should be individualised, taking into account patient choice, followed by counselling of its risks and benefits.
Resectable and borderline resectable disease
Upfront treatment options • Options for upfront resection are likely to be limited due to a lack of capacity and resources. • Where surgery is unavailable, consider upfront SACT or hypofractionated precision RT/CRT. • Where SACT can be used, FOLFIRINOX is preferred and may allow deferral of resection. • For RT, consider 25–35 Gy/5# RT alone or 36–45 Gy/15# CRT with concurrent capecitabine. Adjuvant SACT • Without adjuvant SACT, survival following resection is <10%. • Decisions to give adjuvant treatment are likely to be nuanced and based on a risk–benefit analysis. • Treatment may be deferred by up to 12 weeks following surgery. • The increased effectiveness of combination SACT should be weighed against the increased complications risk.
Locally advanced pancreatic cancer
• For fit patients without significant comorbidities, consider four cycles of modified FOLFORINOX ± consolidation hypofractionated RT/CRT. • The risks of treatment in those aged over 80 years are likely to outweigh any benefit. • For all other patients, consider upfront hypofractionated RT/CRT, with the aim of deferring SACT.
Metastatic disease
First-line treatment • Risks of treatment are likely to outweigh benefits for most patients. • Decisions to treat should be individualised and highly selective. • SACT options include gemcitabine, gemcitabine plus *nab*-paclitaxel or FOLFIRINOX. • Consider early response assessment and limiting duration of SACT where possible. Second-line treatment • Risks of treatment outweigh potential benefits and treatment should not be routinely offered to patients.

*#* fractions, *COVID-19* coronavirus disease 2019, *CRT* chemoradiotherapy, *FOLFIRINOX* 5-fluorouracil, folinic acid, irinotecan and oxaliplatin, *RT* radiotherapy, *SACT* systemic anticancer therapy.

## Diagnosis of pancreatic cancer

With a number of other stakeholders, the British Society of Gastroenterology has published guidance categorising upper gastrointestinal endoscopy as an aerosol-generating procedure and recommending that all elective and non-essential endoscopic procedures should stop.^16^ It is recommended that endoscopic therapy should continue for malignant biliary obstruction, providing an opportunity to retrieve cytology from biliary strictures or in the case of peri-ampullary neoplasm biopsy specimens for some patients prior to self-expanding metal stent insertion. In contrast, 2-week wait cancer referrals and cancer staging endoscopic ultrasound are to be discussed on a case-by-case basis. In instances where histology or cytology cannot be obtained, the multidisciplinary team (MDT) should reach a treatment recommendation based on balancing the risks of inappropriately treating an alternative pathology, such as chronic or autoimmune pancreatitis, against a watch-and-wait approach. Options include proceeding to definitive treatment based on imaging and elevated tumour markers where there is strong suspicion of malignancy or offering treatment where repeat investigations provide evidence for disease progression. Where there is diagnostic uncertainty, patients must be counselled regarding the possibility that they might not have cancer, but would be at risk of developing life-threatening treatment complications, or that in the absence of knowledge of the histological cancer subtype, their treatment might be suboptimal. Percutaneous biopsy may be feasible for more advanced disease, while percutaneous fine-needle aspiration may also have to be considered for localised disease if supported by radiology and pathology expertise.

## Treatment by disease stage

### General principles

There is emerging but relatively low-level evidence that COVID-19 confers additional risk for patients with cancer, although this is not as yet robustly quantified.^7^ Strategies to manage pancreatic cancer should balance this risk and the impact of healthcare resource limitations against the potential benefits of treatment; not least given that significant delays in therapy would ordinarily be precluded by disease biology.^17,18^ Selected approaches will need to adapt to emerging evidence related to COVID-19 and to changes in the availability of key resources. Based on guidance and priority setting from NHS England and the National Institute for Health and Clinical Excellence, systemic anticancer therapy for patients with resectable disease (priority levels 2–4) should be ranked over locally advanced pancreatic cancer (LAPC; priority levels 4 and 5) and metastatic disease (priority levels 4–6), should prioritisation be required (see Supplementary Table 1).^9,11^ A balanced discussion with patients is required to contextualise the known and potential risks of COVID-19 against both the risks of complications from the cancer itself and the potential for complications from anticancer therapy and potential resource limitations. In particular, it must be highlighted that our current ability to mitigate and manage complications associated with pancreatic surgery is predicated on an unlimited access to multidisciplinary services, including physiotherapy, dietetics, nursing, interventional radiology and intensive care.

Where SACT is administered, pragmatic options to mitigate risk include dose modification and the use of prophylactic growth factors and antibiotics. It is also important that all patients adhere to the principles of physical distancing and that they are supported to do so, such as through the use of telephone consultations and remote assessments. In addition, clinical trials and technical development initiatives (robotic surgery) should be stopped in order to minimise resource burden.

In the event of varying regional pressures, particularly during any second peaks of COVID-19, it may be beneficial to refer patients for management in other regions. Where possible, this option should be pursued and facilitated in order to ensure that regional resource limitations do not hinder the provision of optimal care.

### Resectable and borderline resectable disease

Options for upfront resection are likely to be severely limited at the initial height of the COVID-19 pandemic or in the event of recurrent peaks in incidence. Consolidation of surgery in ‘ringfenced’ clean sites has helped to support some surgical capacity during the first COVID-19 peak, although these centres have limited capacity and are likely to be highly selective. Surgery for resectable pancreatic cancer remains the optimal standard of care, and where available should be pursued. Cancer presentation, patient comorbidity, disease severity, regional pandemic burden and regional hospital resources should be considered when selecting patients for surgery. These decisions are likely to remain dynamic and should draw on recommendations from SAGES-AHPBA (Society of American Gastrointestinal and Endoscopic Surgeons-American Hepato-Pancreato-Biliary Association).^19^

Where surgery is unlikely to be available due to a lack of capacity or resources, consider upfront chemotherapy and/or chemoradiotherapy (CRT). Treatment options include SACT (evidence level 2a) and hypofractionated precision RT/CRT, as outlined below, following an informed consent process.^20^ For RT consider a dose of 25–35 Gy/5 fractions (RT alone, dose depending on centre expertise) (evidence level 4) or 36 Gy/15 fractions CRT with concurrent capecitabine (evidence level 1b).^21,22^ For SACT, a combination of 5-fluorouracil, folinic acid, irinotecan and oxaliplatin (FOLFIRINOX) is preferred as the reported median progression-free interval of 15 months could allow deferral of resection in selected patients.^23^ While the magnitude of the additional increase risk conferred by COVID-19 to patients with cancer, particularly those undergoing chemotherapy, is unclear, the risk of death is significantly greater in those with comorbidities and those over 70 years of age.^24,25^ As such, FOLFIRINOX may be most appropriate in patients with a good performance status without significant comorbidities.

Decisions relating to the administration of adjuvant chemotherapy should take into account patient choice, followed by counselling of its risks and benefits. In the absence of adjuvant chemotherapy, 5-year survival for patients who have undergone resection is <10%, compared with over 20% for those who receive adjuvant treatment.^26–29^ For example, in a recent randomised controlled trial, adjuvant FOLFIRINOX delivered 3-year disease-free survival of 39.7% and median overall survival of 54.4 months.^26^ Treatment could also be deferred for up to 12 weeks from surgery (evidence level 1b).^30^ As with neoadjuvant SACT, decision on appropriateness and choice of regimen should be guided by age, comorbidity and potential magnitude of benefit. Nodal status should also be considered given evidence that the outcomes of patients without nodal metastases is more favourable.^28^ The increased effectiveness of combination chemotherapy needs to be balanced with the increased risks of complications, including those relating to COVID-19.

### Locally advanced pancreatic cancer

Patients with LAPC are conventionally managed with upfront chemotherapy, with or without consolidation CRT. The use of upfront hypofractionated (5 fractions, evidence level 2a) or, alternatively, 15 fractions CRT (evidence level 4) may provide lower-risk alternatives and may allow delaying the initiation of or a break in SACT (evidence level 2a).^31^ This approach should, however, be weighed against the risk of early metastatic progression without upfront chemotherapy.^30^ Given the increasing risks of COVID-19 with age, the risks of treatment in those aged over 80 years are likely to outweigh any benefit and no intervention is likely to be the best option for the majority of patients. For fit patients without significant comorbidities, consider four cycles of modified FOLFIRINOX with or without consolidation hypofractionated CRT or five fraction RT alone^23,32^ (evidence level 2a).

### Metastatic disease

The risks of treatment for metastatic disease are likely to outweigh the benefits in many patients as the median improvement in survival is usually <6 months. A decision to initiate palliative chemotherapy should be individualised and highly selective; options for consideration include single-agent gemcitabine, gemcitabine plus *nab*-paclitaxel and FOLFIRINOX in order of increasing efficacy and increasing toxicity (evidence level 1b).^33,34^ In order to mitigate risks, clinicians should consider early response assessment (if radiology capacity allows) to limit duration of chemotherapy. A break from chemotherapy may be considered in patients with low volume disease or those with good disease control (evidence level 5). The limited benefits of second-line chemotherapy outweigh the potential benefits and should not be routinely offered to patients (evidence level 5).

## Hypofractionated radiation approaches

Frequent hospital visits will increase risk of patients contracting COVID-19, therefore conventional CRT (25–30 fractions) should be avoided. Hypofractionated RT (5–15 fractions) reduces footfall, is less immunosuppressive than chemotherapy and the total overall time in hospital is likely to be less than or comparable to patients receiving 3 months of FOLFIRINOX-or gemcitabine-based chemotherapy. Detailed RT delivery guidance document and evidence for their use is available at www.uppergicancer.com. A summary of key points is provided in Table 2.

**Table 2:** Key technical details for the outlined precision hypofractionated radiotherapy approaches.

Regimen	Aspect	Criteria
25–35 Gy/5# RT alone	Indication	• Locally advanced unresectable, or borderline resectable, disease • Recurrent disease • Where patient is unsuitable for surgery
	Investigations	• MRI may be required to aid tumour definition
	Dose to PTV	• 30–35 Gy/5# daily or on alternate days • If no 4D CT, use dose prescription of 25 Gy/5#
	Planning	• IMRT or VMAT
36–45 Gy/15# CRT with concurrent capecitabine	Indication	• Locally advanced disease • Borderline resectable disease
	Investigations	• MRI may be required to aid tumour definition
	Dose to PTV	• Borderline resectable disease: 36 Gy/15# over 3 weeks • Locally advanced disease: 45 Gy/15# over 3 weeks
	Chemotherapy	• Capecitabine 830 mg/m^2^ twice daily on days of radiotherapy
	Planning	• IMRT or VMAT

*CT* computed tomography, *IMRT* intensity-modulated radiation therapy, *MRI* magnetic resonance imaging, *PTV* planning target volume, *RT* radiotherapy, *VMAT* volumetric modulated arc therapy.

### RT alone

#### Dose fractionation: 30 Gy/5 fractions (range 25–35 Gy/5 fractions, daily or alternate day fractionation)

Oncologists who have experience of delivering upper abdominal/pancreatic stereotactic ablative radiotherapy (SABR) could deliver radiation at higher doses of 33–35 Gy/5 fractions using SABR. For those without this expertise, a lower dose of 30 Gy/5 fraction should be considered. Simultaneous integrated boost to tumour/vessel contact (40 Gy) may be considered.^35^

### Chemoradiotherapy

#### Dose fractionation: 36 Gy/15 (preoperative CRT) or 45 Gy/15 fractions (definitive CRT) with capecitabine (830 mg/m^2^ b.d. on days of RT)

This regime should be deliverable by all units with experience in pancreatic RT, the final doses being driven by the normal tissue constraints. A dose of 45–50 Gy/15 fractions is radiobiologically equivalent to conventionally fractionated regimes used in the UK. While the *α*/*β* value for pancreatic adenocarcinoma has not been fully elucidated, it is likely to range between 4 and 10, giving an EQD2 (equivalent dose) of 52.5–61.6 Gy, assuming an *α*/*β* of 4, or of 48.8–55.6 Gy, assuming an *α*/*β* of 10.^36,37^

## Summary

The COVID-19 pandemic poses an unprecedented challenge to the management of patients with cancer; both through a heightened risk of life-threatening infection and through pressure on health services. We have outlined here, based on the best available evidence and UK expert consensus, suggestions for optimising the outcomes of patients with pancreatic cancer. It is vital that decisions are individualised for patients following MDT discussion, and that patients are comprehensively counselled regarding treatment options prior to providing informed consent. Equally, it will be important to evaluate the management options outlined here and clinicians are encouraged to visit www.uppergicancer.com to participate in prospective data collection. Finally, while there is a need to accommodate for the enhanced risks and impact on services from COVID-19, this must not result in a return to the nihilism that has dogged pancreatic cancer for many decades. In these challenging times, compassion and empathy remain key during what is already a frightening period for our patients.

## Supplementary information

Supplementary materials

## Data Availability

Data sharing is not applicable to this article as no datasets were generated or analysed during the current study.
