# Supplementary material for: Considerations for the treatment of pancreatic cancer during the COVID-19 pandemic: the UK consensus position
Source: Br J Cancer. 2020 Jul 8;123(5):709–13. doi: 10.1038/s41416-020-0980-x (PMC7341025; doi:10.1038/s41416-020-0980-x)
Supplement: Supplementary file 1 — Supplementary materials [file 41416_2020_980_MOESM1_ESM.docx]

| **Priority level 1** |
| --- |
| - Curative therapy with a high (>50%) change of success. - Adjuvant (or neo) therapy which adds at least 50% chance of cure to surgery or radiotherapy alone or treatment given at relapse. |
| **Priority level 2** |
| - Curative therapy with an intermediate (20-50%) chance of success. - Adjuvant (or neo) therapy which adds 20-50% chance of cure to surgery or radiotherapy alone or treatment given at relapse. |
| **Priority level 3** |
| - Curative therapy of a low chance (10-20%) of success. - Adjuvant (or neo) therapy which adds 10-20% chance of cure to surgery or radiotherapy alone or treatment given at relapse. - Non-curative therapy with a high (>50%) chance of >1 year of life extension. |
| **Priority level 4** |
| - Curative therapy with a very low (0-10%) chance of success. - Adjuvant (or neo) therapy which adds a less than 10% chance of cure to surgery or radiotherapy alone or treatment given at relapse. - Non-curative therapy with an intermediate (15-50%) chance of >1 year life extension. |
| **Priority level 5** |
| - Non-curative therapy with a high (>50%) chance of palliation/temporary tumour control but <1 year life extension. |
| **Priority level 6** |
| - Non-curative therapy with an intermediate (15-50%) chance of palliation of temporary tumour control and <1 year life extension. |

**SUPPLEMENTARY FILES**

**Supplementary table 1:** A summary of National Health Service (NHS) England guidance for the categorization of patients with cancer into priority groups.(8) NHS England guidance stipulates that patients can be prioritized for treatment according to these categories if services are disrupted.

| **Panel member** | **Specialty** |
| --- | --- |
| Katharine Aitken | Clinical (Radiation) Oncology |
| John Bridgewater | Medical Oncology |
| Pippa Corrie | Medical Oncology |
| Martin Eatock | Medical Oncology |
| Paula Ghaneh | Pancreatic Surgery |
| Rebecca Goody | Clinical (Radiation) Oncology |
| James Good | Clinical (Radiation) Oncology |
| Derek Grose | Clinical (Radiation) Oncology |
| Maria Hawkins | Clinical (Radiation) Oncology |
| Daniel Holyoake | Clinical (Radiation) Oncology |
| Arabella Hunt | Clinical (Radiation) Oncology |
| Nigel Jamieson | Pancreatic Surgery |
| Christopher Jones | Clinical (Radiation) Oncology |
| Somnath Mukherjee | Clinical (Radiation) Oncology |
| Daniel Palmer | Medical Oncology |
| Ganesh Radhakrishna | Clinical (Radiation) Oncology |
| Zahir Soonawalla | Pancreatic Surgery |
| Juan Valle | Medical Oncology |

**Supplementary information:** Roles and relevant expertise of the panel formulating these guidelines.
